# Real-Time Drink Trigger Detection in Free-living Conditions Using Inertial Sensors

**DOI:** 10.3390/s19092145

**Published:** 2019-05-09

**Authors:** Diana Gomes, Inês Sousa

**Affiliations:** Fraunhofer Portugal AICOS, 4200-135 Porto, Portugal; ines.sousa@fraunhofer.pt

**Keywords:** activity recognition, fluid intake monitoring, gesture recognition, inertial sensors

## Abstract

Despite the importance of maintaining an adequate hydration status, water intake is frequently neglected due to the fast pace of people’s lives. For the elderly, poor water intake can be even more concerning, not only due to the damaging impact of dehydration, but also since seniors’ hydration regulation mechanisms tend to be less efficient. This work focuses on the recognition of the pre-drinking hand-to-mouth movement (a drink trigger) with two main objectives: predict the occurrence of drinking events in real-time and free-living conditions, and assess the potential of using this method to trigger an external component for estimating the amount of fluid intake. This shall contribute towards the efficiency of more robust multimodal approaches addressing the problem of water intake monitoring. The system, based on a single inertial measurement unit placed on the forearm, is unobtrusive, user-independent, and lightweight enough for real-time mobile processing. Drinking events outside meal periods were detected with an F-score of 97% in an offline validation with data from 12 users, and 85% in a real-time free-living validation with five other subjects, using a random forest classifier. Our results also reveal that the algorithm first detects the hand-to-mouth movement 0.70 s before the occurrence of the actual sip of the drink, proving that this approach can have further applications and enable more robust and complete fluid intake monitoring solutions.

## 1. Introduction

Hydration has a major impact on human beings’ overall health. While good hydration has been proven to have a positive impact in dealing with several chronic diseases, dehydration has a negative effect on physical and cognitive performance, may cause headaches, potentiate delirium (and delirium presenting as dementia in the elder), and impair gastrointestinal, kidney, heart, and liver functions [1].

Despite its importance, water intake is frequently overlooked, especially at busy times of the day when people do not even realize they are thirsty. Complete fluid intake monitoring solutions should address this problem, with applications ranging from healthcare (e.g., assisting the management of chronic diseases [1]) to occupational therapy, in which typical interventions may include providing tools for proper and safe drinking habits [2].

This work introduces a method that has succeeded in real-time drinking identification both subject-independently and in free-living conditions, relying solely on inertial data from the dominant arm of the users. Additionally, the method can provide useful output to trigger external mechanisms for fluid intake estimation at opportune daily moments, which will contribute towards power efficiency, feasibility, and privacy preservation in increasingly robust multimodal approaches. The main contribution of this study is thus the introduction of a novel approach for drinking recognition, relying on the identification of a single gesture that precedes the sipping event (carrying a liquid-filled container to the mouth), while being fulfilling the following system prerequisites:

- Unobtrusiveness;
- Free-living utilization;
- User-independent;
- Ubiquitous equipment;
- Lightweight and responsive enough for mobile real-time processing.

This paper starts by introducing some related work and discussing the main developments and challenges of current solutions. Then, implemented methods are described, followed by the main results of the algorithm and their discussion. A conclusions section offers some closing remarks.

## 2. Related Work

This literature review focuses on two distinct topics: detection of drinking periods, especially in free-living conditions, and amount of fluid intake estimation. A subsection discusses some difficulties identified in the surveyed works, highlighting the challenges and opportunities in this research field.

### 2.1. Drinking Detection

Some human activity recognition works have used inertial sensing to recognize drinking from among a range of activities. In [3], the authors recognized a total of 20 different high-level daily activities with an overall accuracy of 84%. Their system was composed of five sensing devices, and the authors did not distinguish eating from drinking, but considered them in the same class instead. The authors of [4] also conducted remarkable work in complex activity recognition from a range of activities in which drinking was included, using inertial sensing and taking activities as a whole instead of splitting them into gestures. Following this premise, the authors came to the conclusion that the larger the windows, the better the complex activity classification results. Gesture spotting was used in several other works. In [5], drinking was distinguished from typical eating-related gestures (eating with cutlery, spoon or hand-held) with 83% recall and 88% precision. While studying the importance of sequential dependency between gestures, [6] concluded that in the course of a meal, drinking was the movement in which sequential dependence was least important (compared to using utensils, resting, biting, and a rejection class). Nonetheless, their best approach was able to distinguish drinking with 82% accuracy using a sixth-order hidden Markov model (HMM). More recently, another approach to the problem of food and drink intake recognition was brought forward by [7], in which five gestures were distinguished: drinking, eating with one’s hand, eating with a fork, eating with a spoon, or cutting. The method relied on two inertial measurement unit (IMU) devices, one placed on the waist to provide data concerning the user’s ambulatory status and the second placed on the wrist to enable gesture recognition. They finally achieved an eating and drinking gesture recognition rate of 99%.

Detecting drinking from among pre-defined sets of activities, whether these are simple gestures or more complex movements, may, however, be insufficient for full application within this work’s purpose. These works, while providing some information about the nature of the drinking movement and how different it might be from gestures we would perceive as similar, do not pursue drinking recognition in real-time or free-living conditions. For example, not defining a rejection class able to hold all non-drinking activities is a drawback of some approaches for free-living applications. Moreover, some of the aforementioned works rely on processes with a high computational load, which hinders their suitability for mobile processing, and rely on multiple sensing devices, a negative aspect in terms of a system’s usability and obtrusiveness. In [8], however, the authors conducted an experiment in a controlled environment and achieved 84% recall and 94% precision in recognizing drinking motions from a continuous data stream, with only one inertial sensor placed on the wrist. Even though they tried to encourage natural movements, one can argue that some daily gestures that occur spontaneously and may resemble drinking, in the course of a day, might not have happened. This can impact the precision of the algorithm in a free-living conditions validation. Moreover, their method was user-specific, meaning the authors did not face the challenges of inter-subject variability. A previous study had already tried to distinguish drinking from eating movements in [9], but this work focused solely in distinguishing gestures, i.e., it did not aim to distinguish drinking from other activities performed daily.

Besides inertial sensing, some other data sources have also been explored for drinking detection. The work of [10] relied on radio-frequency identification (RFID) technology to instrument a cup and detect drinking events that involved that container. On the plus side, this technology does not require batteries/charging, but it is restricted to a single location, usually the home of the user, where the antenna system is placed. Their recognition of drinking episodes for younger and older subjects led to F-scores of 87% and 79%, respectively. The study aimed to mimic free-living conditions but was still performed under a controlled environment. Some other works have also focused on detecting drinking (and eating) movements in fixed settings, such as the dinning table, with smart-tagged objects [11]. Audio has also been explored by several authors for drinking detection, frequently unveiling promising results. In [12], the authors were able to recognize drinking activity with an F-score of 78.5%, out of a range of activities that involve throat-related noises (e.g., cough, deep breath, eating, speaking). Their system, BodyScope, consisted in the combination of a Bluetooth headset with embedded microphone and a stethoscope chestpiece. A different wearable acoustic system, BodyBeat, was proposed by [13]. It aimed at recognizing non-speech body sounds with a microphone placed on the throat and held by a neckpiece. Drinking was identified with 72% recall and 57% precision.

### 2.2. Fluid Intake Estimation

Detecting whether a user has taken a drink is not the only challenge in creating a complete drinking reminder and assistance tool; it is also important to estimate the amount of ingested fluid. The aforementioned work of [8] also established further research objectives aimed at estimating the fluid level and container types solely with inertial sensing. Even though all experiments took place in a controlled environment, limited recognition rates of 75% and 72% for container type and fluid level, respectively, were achieved with a C4.5 Decision Tree classifier.

Multimodal approaches appear to be more common to estimate the amount of fluid intake. The Playful Bottle system was developed to monitor drinking events and persuade the users to ingest more water while working in an office [14]. The system consists in attaching a smartphone to a specially designed mug (transparent and striped), and using its camera and accelerometer sensors to provide data for a vision/motion-based water intake tracker. Acceleration data is used to decide whether a drinking event took place; five seconds after a drinking event, the camera is activated to infer the difference in water level based on the camera/striped mug system. In case of refill, the user must click the refill button in the application in order to set a new water level. Their method succeeded in estimating water intake with an error rate of just 3.86% for tests conducted with 16 subjects. However, the method for recognition of drinking events could still be improved, since they report 21.1% false positive detections. In [15], the authors proposed a novel method for automatic food/drink intake detection with type and weight estimation. The system used audio (external and internal ear microphones) and inertial (smartwatches on both wrists and Google Glass) data annotated for several activities (namely intake, chew, and swallow). They assessed weight estimation performance by computing the relative mean absolute percentage error as the mean of the absolute value of the difference between real and estimated weight for each intake over mean intake size for that food/liquid, and reported a weight estimation error of 47.2% for drinking events.

Other works have also used sound data to detect swallowing. Even though these works have particularly focused on eating activities, some of them also distinguish drinking from eating, like AutoDietary, which recognizes liquid intake events with an accuracy of 97.6% [16]. Using swallow detection methods to try to estimate the amount of ingested fluid is a path explored by few works, however. Audio-based methods are also associated with privacy concerns due to the occasional sensitivity of sound data, and require special attention to power consumption as the high sampling rate often required makes this approach less power efficient than other sensing modalities.

### 2.3. Challenges and Opportunities

There are still several challenges to overcome in drinking detection. Several of the aforementioned works report modest values of precision, since false positive occurrences remain fairly frequent, regardless of the sensing modality in use. Trying to tackle this difficulty has led to more complex systems, most of which suffer from acceptance issues due to their size, appearance, obtrusiveness, impracticality, or poor power efficiency, leading to a potential lack of adherence by target users. The simplicity, unobtrusiveness and portability of inertial sensing-based systems remain a strong advantage of such systems and motivates further research to explore its full potential.

Among the surveyed solutions to measure fluid intake, multimodal sensing approaches have often been more successful than others (e.g., audio and inertial data sources). This leaves a gap and a need for techniques that aim for power consumption optimization in multisensing drinking recognition methods. At the same time, the advances in gesture spotting research have shown that drinking-related gestures are in fact distinguishable from eating-related gestures, regardless of how similar they may seem. All this can be combined in order to motivate the pursuit of complete drinking characterization solutions further focused on more ambitious goals such as non-invasive fluid intake estimation. Identifying the pre-drinking stage (or a drink *trigger*) with a ubiquitous and low power consumption device (an IMU from a smartwatch or smartband, for example) could be a first step towards a robust drink-monitoring system since further sensing devices could be opportunely activated solely when a drinking event is about to occur, which enables a power-efficient and accurate estimation of fluid intake through multimodal approaches.

## 3. Methodology

### 3.1. Data Collection and Annotation

**Figure 1:**
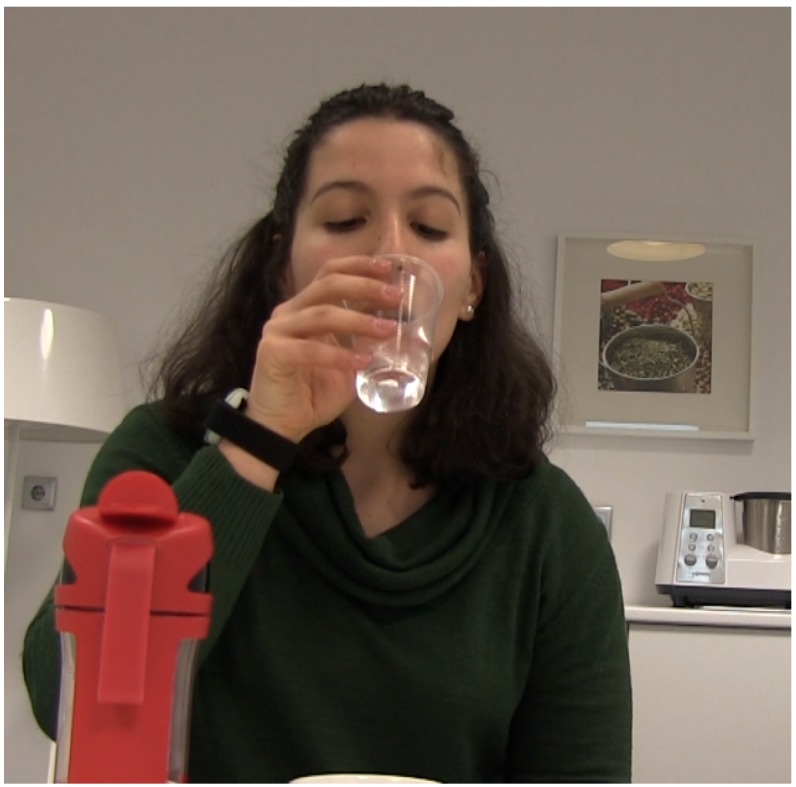
Example of typical IMU placement on the forearm and fluid container.

Data was collected from volunteer participants who wore a Bosch BMI-160 IMU sensing device from a proprietary hardware platform, the Pandlets [17], enclosed in an in-house-built case. The IMU was placed on the dominant forearm just above the wrist, as depicted in Figure 1. The IMU was placed identically on each individual, with the direction of the *x*-axis aligned with the arm and pointing towards the hand, the *y*-axis perpendicular to the latter in the hand–arm system plane (in a resting position, pointing towards the body), and the *z*-axis perpendicular to *x* and *y*, pointing in the direction of the back of the hand. Data from the accelerometer and gyroscope were sampled at 100 Hz, as is usual for human gesture recognition, and streamed by Bluetooth Low Energy to a smartphone recording application.

#### 3.1.1. Dataset 1

Dataset 1 was planned to address the need to obtain inertial data from activities involving hand motion, including handling objects, movements towards the mouth, face or head, and others. Eating activities were deliberately left out of this dataset because among the variety of gestures performed during meals, one can also find situations in which fluid containers (including not only cups, mugs, or bottles, but also, for example, spoons) are handled and carried to the mouth. In this work, our goal was to focus on the recognition of the pre-drinking movement, i.e., the hand-to-mouth (HtM) gesture, as a way of predicting that a drinking event is about to occur. As such, considering the entire eating activity as part of the rejection class would be misleading for the training of a classifier that aims to predict the occurrence of that single gesture, which is most likely also present among the miscellaneous meal-related events. This situation is further discussed in Section 5.

A total of 12 subjects (23.7 ± 2.3 years old) gave their informed consent and participated voluntarily in a data collection session. Each of them was asked to drink several times from different containers and perform a set of other activities that involved hand motions, namely other hand-to-mouth movements, under different whole-body activity contexts (two postures and walking). All activities were performed sequentially and later annotated using video data. Table 1 describes the activities performed by the volunteers, including the number of times each one was repeated.

**Figure 2:**
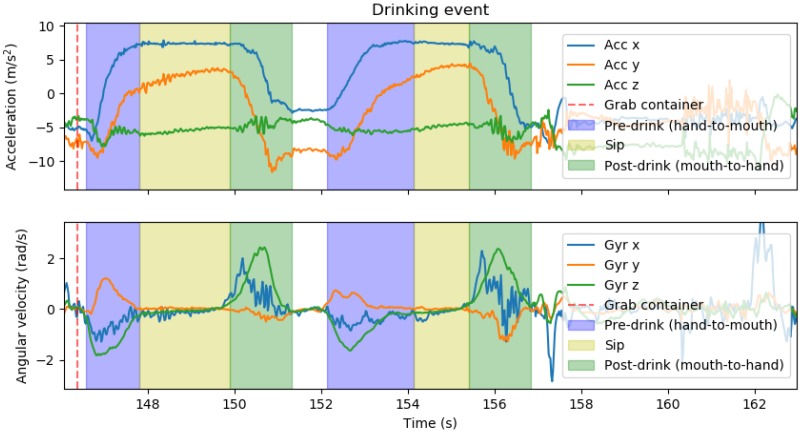
Annotated drinking event. Accelerometer (Acc) and gyroscope (Gyr) signals are shown.

Video and inertial data were synchronized by capturing the moment when the application started data collection, i.e., the moment the user pressed its start button, and setting that moment as $t_{0}=0$. Video was annotated with frame precision using the Kinovea software, an open-source tool for video analysis that supports several useful functionalities, such as the possibility of annotating video frames and exporting the final result with its associated timestamp [18]. Each drinking event was annotated for several moments/intervals: grab the container (when it occurred), take it to the mouth (pre-drinking hand-to-mouth movement), have a sip, go back to a resting position (post-drinking mouth-to-hand movement). For all other activities, only start and end moments were annotated. Figure 2 shows an example of an annotated drinking event.

The dataset comprised a total of 312 drinking movements and 216 other activities involving hand gestures. Considering the goal of detecting the hand-to-mouth movement while handling a liquid-filled container (HtM), binary target classes were defined—HtM and not HtM—and used for all classification purposes in this work.

#### 3.1.2. Dataset 2

Five volunteers (24.4 ± 2.1 years old) that did not participate in the previous data collection were asked, after giving their informed consent, to place an IMU device on their dominant forearm and use it unrestrictedly during 3 to 5 h, according to their availability. During that time, the participants were asked to behave normally and drink water at will, as frequently as possible. Each participant was given a smartphone to which inertial data was streamed and processed by an application especially developed for that purpose. This processing corresponded to the flow of operations of the developed algorithm and included the deployment of a random forest classifier previously trained using data from Dataset 1 (see more details in the following subsections).

Whenever a drink trigger was detected, the phone would vibrate to alert the user. This alert enabled the annotation of events by the user. As such, the only interactions the users had with the phone corresponded to moments when:

1. HtM was detected but no drinking action took place: the user should add an FP occurrence by clicking the respective button in the application;
2. HtM was not detected when a drinking action took place: the user should add an FN occurrence by clicking the respective button in the application.

### 3.2. Feature Extraction and Selection

Accelerometer and gyroscope data were segmented in fixed-length windows of 1 s with 50% overlap, meaning that a new window of 1 s was constituted and processed every half a second. In this way, the training set was finally composed of 1.034 instances of the positive class and 11.526 instances of the negative class.

Ten statistical time-domain features were implemented: mean, standard deviation, maximum, minimum, variance, covariance, correlation, mean absolute deviation, kurtosis, and skewness. Covariance and correlation were computed from the signals of each sensor’s axis in combinations of two. The remaining were computed from 10 different input signals: each of the 3 axes of the accelerometer and gyroscope, magnitude of acceleration, magnitude of angular velocity, and pitch and roll angles. Pitch and roll were obtained through the sensor fusion algorithm based on gradient descent optimization proposed by [19]. No frequency-domain features were implemented to reduce computational load. This process led to an initial feature vector of size 94.

Then, a feature selection step took place using the intrinsic capabilities of random forest classifiers for feature ranking based on the computation of Gini impurity [20]. Models were trained with the extracted features, to which binary HtM vs. not HtM labels were assigned. Features that allowed the foremost decisions within the trained classifier were iteratively selected and the remaining eliminated, until a negative impact in the classification results was verified. This led to a final selection, after 3 iterations, of the 10 following features, in order of importance:

1. Maximum of gyroscope’s *z*-axis
2. Mean of gyroscope’s *z*-axis
3. Correlation of accelerometer’s *x* and *y* axis
4. Maximum of gyroscope’s *x*-axis
5. Minimum of gyroscope’s *z*-axis
6. Standard deviation of gyroscope’s *x*-axis
7. Variance of gyroscope’s *x*-axis
8. Mean of accelerometer’s *x*-axis
9. Maximum of accelerometer’s *x*-axis
10. Covariance of accelerometer’s *x* and *y* axis

### 3.3. Classification

The random forest classifier [21] was selected for the purpose of identifying HtM gestures. During training, each sample fed to the classifier was associated with a binary target class, as previously described. The implementation of random forest from scikit-learn [22], in Python language, was used to conceive the classification model, and all offline validation tests were conducted based on this model. The algorithm was also implemented in Java language, with the trained classifier exported to Java using sklearn-porter [23]. An Android application was then developed to support the real-time validation of the algorithm, with simplified portability and usability, with the aim of enabling a more natural utilization of the system in free-living conditions during the validation stage.

### 3.4. Validation

Two different validation protocols were created to assess the performance of the proposed algorithm. The first consisted of an offline process, using Dataset 1. Inter-subject variability was accounted for by performing leave-one-subject-out (LOSO) validation. Performance metrics that referred to the ability to identify an HtM movement before the occurrence of a true drinking event were extracted (true positives (TP), false positives (FP), false negatives (FN), recall, precision, F-score). Since we wanted to detect a drink trigger, i.e., a single timestamp before the drinking event rather than an interval, TP, FP, and FN occurrences were not directly extracted from the window-based metric system; instead, the trigger timestamp was set to correspond to the end of the first time-window classified as HtM in a row, and a verification of whether it belonged to a true HtM period was implemented. This means that metrics were computed over the universe of HtM movements performed in the entire dataset, rather than with a window-based methodology. This enabled the definition of all metrics with respect to the occurrence of a single timestamp, thus facilitating comparison with the results of the real-time validation (of trigger-based output). Further metrics were computed in order to completely characterize HtM detection in time, taking advantage of the meticulous dataset annotation, and to assess the suitability of the method as a trigger for further sensing mechanisms. In particular, the time interval between the start of the HtM movement and its detection (trigger delay) and the time between detection and the start of the sip (time to sip) were computed. Then, a free-living real-time validation study was conducted to foresee the potential of the method when facing the challenges of real-world unrestricted utilization. Dataset 2 provided the basis for this study.

## 4. Results

The results obtained following the two distinct validation protocols are presented in Table 2. Due to the nature of the protocols, made clear in the previous section, trigger delay and time to sip metrics could only be estimated for the offline validation. The following subsections explore the results for each implemented validation protocol separately.

### 4.1. Offline Validation

The offline validation with the attained dataset resulted in the accounting of false detections and missed detections, i.e., true drinking events that were not considered as such. Furthermore, considering the goal of being able to interact with and trigger fluid intake estimation mechanisms, the delay of the trigger (time from the moment the HtM movement starts to its actual detection) and how long before the actual sip it occurs were also computed. Leave-one-subject-out validation was used to preserve subject independence.

The results reported in Table 2 evidence an adequate detection of drinking HtM gestures and indicate that it is in fact possible to distinguish a gesture that involves carrying a liquid-filled container to the mouth from other hand-to-mouth gestures (involving or not involving other objects). Only 8 movements were mistakenly perceived as drinking instances, despite the fact that (1) all of the activities in Dataset 1 that belonged to the rejection class involved hand motion; (2) three higher level, whole-body activities were tested; and (3) the volunteers had some opportunity to improvise, which led to more natural and variable movements. With z a recall of 97%, the high precision obtained is, thus, really promising towards an application in free-living conditions.

HtM movements have a short duration; in this dataset, volunteers took 1.68 ± 0.81 s (average ± standard deviation) to perform them. Since time windows of 1 s were processed every 0.5 s, this would mean that each movement should be captured by two to four windows. The detection of such a limited timespan could be impaired by the effects of boundary situations (associated with the subjectivity of the annotation process and/or inherent to the implemented windowing method) and, thus, be unsuitable as a trigger for external mechanisms. The trigger delay and time to sip metrics reveal, however, that this is not the case, as triggers occur, on average, 0.70 s before the beginning of the sipping event, less than a second after the movement starts.

### 4.2. Real-Time Validation

The results for the real-time validation process are associated with a much looser protocol than the previous validation, as there were no movement restrictions, videotaping, or external people supervising the process. The smartphone–IMU band system was familiar to the volunteers, who interacted with the system without difficulty. Therefore, it can be assumed that movements were performed as naturally as possible, constituting a higher challenge to the classifier and leading to a small decrease in the performance metrics, as expected. Nonetheless, HtM movements preceding a drinking event were detected with an F-score of 85%, leading to the conclusion that FP and FN occurrences are balanced and rare, even in free-living real-world conditions.

This validation procedure also accounted for the problems that can occur in the case of real-time prolonged use of systems (e.g., losses of connection, delays, sensor asynchronism) and proved to respond adequately to such problems. Furthermore, it proved that the system is lightweight enough for real-time processing of streamed data every 0.5 s, an important prerequisite for portable systems suitable for real-world utilization.

## 5. Discussion

The pursuit of pre-drinking stage identification was encouraged by the fact that several works have shown that drinking gestures are in fact distinguishable from other gestures that are perceived as similar at first glance. This has been confirmed by works focusing on the distinction between meal-related movements [5,6,7]. Even though Dataset 1 did not include eating-related gestures, this dataset provided an adequate testbed for analyzing this premise, since it comprised data from several activities involving hand motions, including hand-to-mouth movements. Moreover, volunteers reported eating activities, namely snack breaks, while acquiring data for Dataset 2, and did not report the occurrence of FPs during that activity. This indicates that no particular confusion between eating- and drinking-related movements was noticed by the users during the real-time validation. Thus, the overall results support the observation that hand-to-mouth gestures while carrying a fluid container can be distinguished from other hand-to-mouth gestures involving different types of objects or no objects. Nonetheless, and despite being outside of the scope of this work, the definition of drinking as a sub-activity of meal/eating activities is very common and should be specifically addressed in future work.

If the application of the proposed method is limited to drinking event detection, one can consider the work of [8,10], which also classified drinking events against a rejection class (binary drinking classification, instead of multi-class). The subject-dependent approach of [8] recognized drinking episodes with an F-score of 89%, while the best reported performance of [10] is an F-score of 87%, in young subjects. These validations, however, were performed under controlled environments. The results from our subject-independent offline validation, also performed in a controlled environment, reveal a considerably higher F-score of 97%. However, this comparison might not be fair as the activities with representation in the testing sets were not the same. Our real-time free-living validation revealed an F-score of 85%, only slightly inferior to that of the aforementioned works. The variety of movements performed under free-living conditions usually has a negative impact on the performance of activity recognition methods, so it would be necessary to test the algorithms of [8,10] under the same conditions to come to a fair conclusion. As such, the proposed method brings promising results and an advance towards increased robustness in drinking monitoring systems.

By reporting an overall appropriate performance in drinking recognition, this work shows that it is possible to identify the HtM gesture with effective and well-established methods of low-to-moderate complexity, thus enabling its successful mobile implementation with real-time processing. The feasibility of a power-efficient and practical multimodal solution for fluid intake monitoring based on the proposed system was also assessed. Since the algorithm first detects the event 0.70 s before the sip, it should be safe to assume that it can trigger a response mechanism for fluid intake estimation. Interacting with audio-based methodologies, for example, may involve an even bigger time-lapse if throat noises are being monitored, since gulping occurs after sipping.

The obtained dataset aimed to represent as many types of hand-to-mouth movements as possible, but it did not guarantee that all existing contexts or movements were accounted for, or that they were performed naturally. This can be considered a limitation of the study. While we tried to tackle this by performing a free-living validation, extending this validation with more test subjects could reveal more about the potential of the algorithm. In particular, it could be interesting to add data samples from elderly subjects, as gestures may be performed differently or at a slower pace. Even so, these preliminary results are considered very promising and an extension of the state-of-the-art, being the outcome of a free-living and real-time validation protocol with online mobile data processing. Another limitation, however, is the fact that the real-time validation relied on unsupervised user-logged data. This method may be more prone to error, but it also increased user comfort while using the system. Thus, since this protocol was created with the fundamental purpose of capturing the most natural free-living movements, this approach was still deemed preferable.

## 6. Conclusions

This work presented a novel approach to address the problem of unobtrusive fluid intake monitoring in free-living conditions. It shows that it is possible to rely on the identification of the hand-to-mouth movement before sipping a drink to predict the occurrence of a drinking event, even though several other hand-to-mouth movements which do not involve drinking may take place during the day.

Our approach stands out in terms of the state-of-the-art by implementing efficient methods with the goal of detecting an activity-predictive gesture in real-time and real-world conditions. The occurrence of a drinking event is thus predicted rather than detected upon or after fluid intake. The predictive nature of the method will contribute towards the efficiency of more robust solutions for fluid intake estimation, being suitable for the triggering of external sensing mechanisms 0.70 s (on average) before the sipping event.

Drinking activity has usually been studied as a part of more general automatic dietary monitoring. However, our work should bring awareness to the specificity of drinking-related gestures, especially since they involve handling fluid-filled containers. Therefore, in future work we plan to focus in the drinking activity, not only to improve current methods, but also working towards the development of complete solutions for water intake management, since the applications of hydration monitoring can extend those of automatic dietary journaling, namely in healthcare and occupational therapy.

## Figures and Tables

**Table 1:** Activities performed by each subject during data collection. *N*x is the number of repetitions.

Label	Whole-Body Activity	Description	*N*x
Drink	Sit	Grab cup and drink	2
		Drink from cup	2
		Grab mug and drink	2
		Drink from mug	2
		Grab bottle and drink	2
		Drink from bottle	2
	Stand	Grab cup and drink	2
		Drink from cup	2
		Grab mug and drink	2
		Drink from mug	2
		Grab bottle and drink	2
		Drink from bottle	2
	Walk	Drink from bottle	2
Other	Sit	Pick up a call	1
		Fix hair	1
		Scratch face	1
		Move hands, talking	1
		Cough	1
		Hold head with hand	1
		Bite nails	1
		Pick object up from floor	1
	Stand	Pick up a call	1
		Fix hair	1
		Scratch face	1
		Move hands, talking	1
		Cough	1
		Hold head with hand	1
		Bite nails	1
		Pick object from floor	1
	Walk	Pick up a call	1
		Scratch face	1
		Cough	1

**Table 2:** Drink trigger detection results. LOSO: Leave-One-Subject-Out.

Dataset ID	Validation Protocol	Number of Triggers				Recall	Precision	F-Score	Trigger Delay (s)	Time to Sip (s)
		Total	TP	FP	FN					
1	Offline; LOSO	312	303	8	9	0.97	0.97	0.97	0.97 ± 0.56	0.70 ± 0.67
2	Real-time; free-living	164	113	27	24	0.85	0.84	0.85	n.a.	n.a.
